# Quantification of Caffeine and Chlorogenic Acid in Green and Roasted Coffee Samples Using HPLC-DAD and Evaluation of the Effect of Degree of Roasting on Their Levels

**DOI:** 10.3390/molecules26247502

**Published:** 2021-12-11

**Authors:** Shady Awwad, Reem Issa, Lilian Alnsour, Dima Albals, Idrees Al-Momani

**Affiliations:** 1 Department of Pharmaceutical Chemistry and Pharmacognosy, Applied Science Private University, Amman 11931, Jordan; 2 Department of Pharmaceutical Sciences, Pharmacological and Diagnostic Research Center (PDRC), Faculty of Pharmacy, Al-Ahliyya Amman University, Amman 19328, Jordan; l.alnsour@ammanu.edu.jo; 3 Department of Medicinal Chemistry and Pharmacognosy, Faculty of Pharmacy, Yarmouk University, Irbid 21163, Jordan; dimabals@yu.edu.jo; 4 Department of Chemistry, Faculty of Science, Yarmouk University, Irbid 21163, Jordan; imomani@yu.edu.jo

**Keywords:** coffee, caffeine, chlorogenic acid, high-performance liquid chromatography (HPLC), quantification, extraction

## Abstract

Chlorogenic acid and caffeine are among the important components in coffee beans, determining the taste and aroma. In addition, phenols and antioxidants content possess vital health values. The main aim of this study is to determine the levels of caffeine and chlorogenic acid in several coffee samples of different origins and degrees of roasting. The coffee samples were extracted using hot water. The levels of caffeine and chlorogenic acid were quantified using high-performance liquid chromatography (HPLC) equipped with a diode array detector, a reverse phase system, and an ODS column (C18). Total phenol and antioxidant contents were previously determined for the same samples. The results showed that the highest content of caffeine was found in the medium roasted coffee (203.63 mg/L), and the highest content of chlorogenic acid content was found in the green coffee (543.23 mg/L). The results demonstrated a negative correlation between the chlorogenic acid levels with the degree of roasting, while it showed a positive correlation between the caffeine levels with the degree of roasting till a certain point where the levels dropped in the dark roasted coffee. The origin of coffee samples did not show any effect on any of the measured variables. Antioxidant effects of coffee samples were largely determined by chlorogenic acid content.

## 1. Introduction

Chlorogenic acids (CGAs), a group of phenolic esters of caffeic and ferulic acids, are found in many plants, fruits, and vegetables. They are reported to be found in high concentrations in coffee, as high as 70–350 mg per cup of coffee [1]. More than 80 different CGAs were detected in green coffee beans, with 5-caffeoylquinic acid being the most abundant [2].

The biological importance of CGAs was studied throughout the years, demonstrating high antiviral, antidiabetic, antioxidant, and neuroprotective potential [1,2,3]. A study by Zuo et al. [1] recently reported a significant anti-hepatitis B virus activity of CGA, caffeic acid, and quinic acid; while Tunnicliffe et al. [3] have reported a direct effect of CGAs on slowing intestinal glucose absorption and inhibiting hepatic glucose output, thus having an important role in regulating glucose metabolism. CGAs also exhibit antioxidant and antiaging effects by enhancing cell proliferation, lowering lipid peroxidation, and accelerating wound healing, according to Chiang et al. [4].

A recent study by Tang et al. [5] demonstrated a strong correlation between antioxidant capacity and total phenolic compounds in the Pitahaya fruit peel, highlighting the contribution of each phenolic compound (including CGAs) to overall antioxidant capacity. Results have shown that CGA, one of the major phenolic compounds found, did not contribute significantly to the antioxidant activity as an individual compound. However, the copresence of CGA with other compounds in the extract may have an antagonistic effect, which may explain the low contribution of CGA to overall antioxidant activity in wild rice samples [6]. In another study done on *Lonicerae Japonicae Flos*, a Chinese traditional herb, it was found that CGA possesses strong antioxidant activity, which was correlated to total phenolic content. Moreover, the main contributor was CGA [7].

In addition, the degree of roasting and geographical origin of coffee beans were shown to affect the CGA content. The higher the roasting degree, the lower the CGA content as it is decomposed with exposure to heat [8].

Caffeine is another important component of coffee beans, which belongs to the methylxanthine class. It is a bioactive compound that has CNS stimulatory effects, causing alertness and mood enhancement. A single cup of coffee may contain between 95–330 mg of caffeine. Most evidence suggests that caffeine intake of 400 mg per day or less has beneficial effects on the body. For example, it may lower the risk of Parkinson’s and Alzheimer’s disease, as well as lowering the risk of type 2 diabetes [9].

The concentration of caffeine in coffee varies depending on plant species, genetic traits, agricultural practices, storage conditions, roasting degree, and brewing method [9]. Fuller and Rao [9] claim that cold brew coffee made with medium roast had higher concentrations of caffeine and 3-CGA than their dark roast counterparts. Moreover, cold brew coarse grind samples had a higher concentration of caffeine than hot brew samples. Alqarni et al. [10] confirm that dark roast coffee has lower caffeine and antioxidant capacity compared to raw coffee.

The present work aimed to determine the CGA and caffeine content in *Coffea arabica* samples consumed in Jordan, of various geographical origins and degrees of roasting, and to compare the outcomes with total phenolic and antioxidant contents of the samples, to determine if there is any correlation present.

## 2. Results

### 2.1. Caffeine and CGA Content in Coffee Beans Based on Roasting Degree

In this study, coffee samples were extracted and analyzed to assess the levels of caffeine and CGA using HPLC-DAD. The experimental results for the caffeine and CGAs analysis in coffee samples are presented in Supplementary Table S1. The HPLC-DAD chromatograms for the caffeine and CGAs (standard solution and coffee extract samples) are also presented in the same Supplementary Figures S1–S4).

The concentrations of caffeine and CGA in percentage (C, %) and mg/L in green and roasted coffee beans based on roasting degree are reported in Table 1. The table shows that green coffee beans contain the highest CGA, but the lowest caffeine content. The CGA content is shown to decrease with increasing roasting degree. The caffeine content was increased from green beans through medium roasted coffee beans. On the contrary, dark coffee beans were shown to contain the lowest content of CGA, with a moderate content of caffeine. The statistical analysis for all coffee samples of different roasting degrees (green, light, medium, and dark) indicated that no significant difference (*p* ≥ 0.05) in the concentrations of caffeine in the coffee samples was detected. On the other hand, the results of the CGAs concentrations showed a significant difference (*p* < 0.05) for the four roasting degrees of coffee samples.

### 2.2. Caffeine and CGA Content in Coffee Beans from Different Geographical Origin

**Figure 1:**
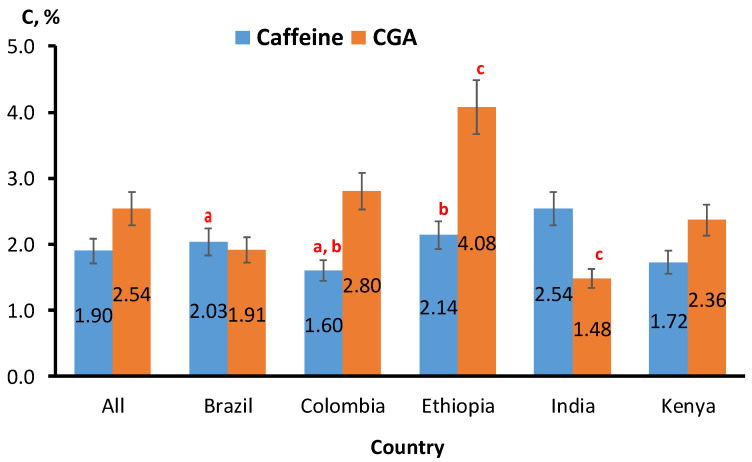
The caffeine and CGA content (C, %) in coffee beans from different geographical origin (different roasting degrees). More details concerning the number of samples for each origin are available in the Supplementary Table S1. Bars labeled by the letters ^a,b,c^ are statistically significant (*p* < 0.05).

The concentrations of caffeine and CGA in percentage (C, %) in green and roasted coffee beans from different countries are shown in Figure 1. It revealed that they is no correlation with the geographical origin and the content of these components.

The statistical analysis showed that different coffee bean origins have no significant difference (*p* ≥ 0.05) on the concentrations of caffeine in the coffee samples, except for coffee samples originating from Brazil and Columbia (*p* < 0.05). Similarly, no significant difference (*p* ≥ 0.05) on the concentrations of CGAs in the same coffee samples was found, except for coffee samples originating from Ethiopia and India (*p* < 0.05).

**Figure 2:**
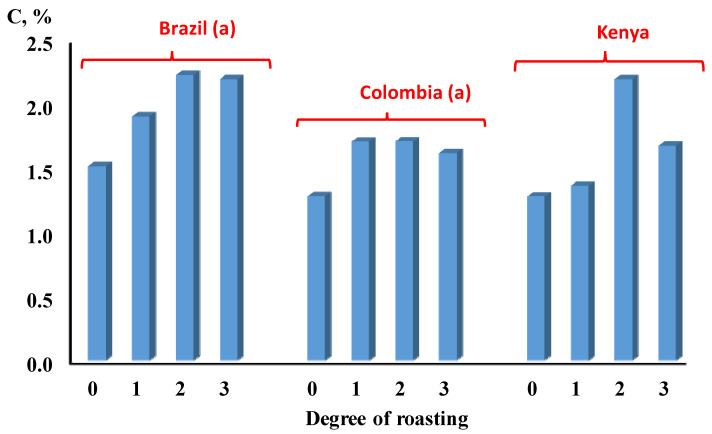
The effect of degree of roasting on the concentration of caffeine in coffee beans from different sources. (0 = green, 1 = light roasting, 2 = medium roasting, 3 = dark roasting). Bars labeled by the letter ^a^ are statistically significant (*p* < 0.05).

The levels of caffeine extracted from the coffee samples showed a positive correlation with the degree of roasting temperature, but it fell short in the dark roast coffee as indicated in Figure 2. In this figure, the levels of caffeine increased, as the temperature of roasting increased, reaching its highest level in the medium roast coffee as can be seen in different types of coffee origins.

**Figure 3:**
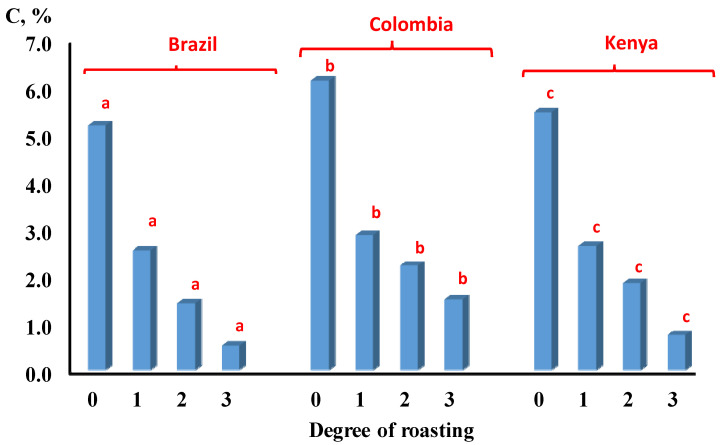
The effect of degree of roasting on the concentration of CGA in coffee beans from different sources. (0 = green, 1 = light roasting, 2 = medium roasting, 3 = dark roasting. Bars labeled by the letters ^a,b,c^ are statistically significant (*p* < 0.05).

However, the levels of CGAs extracted from the coffee samples showed a negative correlation with the degree of roasting as indicated in Figure 3. In this figure, the levels of CGAs decreased, as the roasting degree increased in all different types of coffee origins.

### 2.3. Correlation between the Effect of Roasting Degree with Caffeine, CGA, GAE, and TEAC Contents

Results from our previous unpublished study entitled “Quantification of total phenols and antioxidants in Green and Roasted Coffee Samples of Different Origins and Evaluation of the Effect of Degree of Roasting on Their Levels” using the same coffee samples were used to investigate the effect of degree of roasting on the concentration of caffeine, CGA, total phenols equivalent to gallic acid (GAE) and antioxidant equivalent to Trolox (TEAC) in coffee beans from different sources. These parameters would collectively reflect the health benefits of drinking coffee as a common beverage, and may also contribute to the choice of coffee type that individuals would make, in addition to its known influence on the taste of coffee.

**Figure 4:**
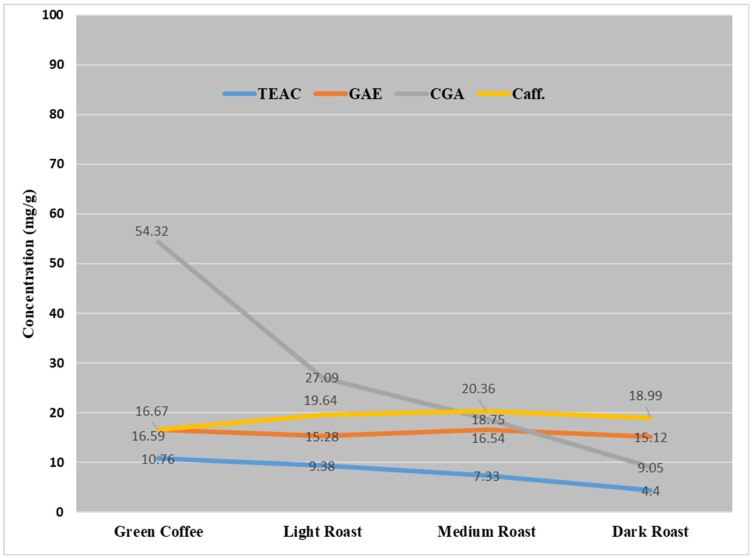
The effect of degree of roasting on the concentration of caffeine, CGA, GAE (total phenols) and TEAC (antioxidants) in coffee beans from different sources.

Figure 4 shows that the largest effect of the roasting degree was on the content of CGA and TEAC, which showed a sharp reduction in their content in green beans through the roasted samples. On the contrary, the effect of roasting degree on caffeine content showed a slow increase from green samples to light and medium roasted samples, followed by a reduction with its content upon the dark roasting level. The content of GAE showed a fluctuation in its content among different roasting degrees.

## 3. Discussion

This research was carried out to evaluate the contents of caffeine and CGAs using the HPLC-DAD method in several coffee samples of different origins, which were purchased from the Jordanian local market. The research was also conducted to investigate the influence of the degree of roasting on the levels of caffeine and CGA. The extraction and quantification methods proved to be simple, efficient, sensitive, accurate, precise, and linear in the specified range.

The results of this research demonstrated that the medium roasted coffee beans have the highest concentration of caffeine, while the green coffee has the highest concentrations of CGA in the studied coffee samples, which agrees with the results of most literature cited. A study by Macheiner et al. [11] investigated green coffee infusion as a source of caffeine and CGA. The results indicated that the levels of CGAs decreases, and these compounds will undergo thermal degradation as the temperature of roasting increases. The loss of CGA levels during the roasting process of coffee beans has been previously reported in several studies. It was demonstrated that the use of high temperature during the process of roasting can lead to the breaking of the carbon-carbon bonds of the CGA structure, which would result in thermal degradation and isomerization of the CGAs structures [12]. Results for caffeine in green coffee infusion reached from 113 to 188 mg/L in *C. arabica* infusions, and for CGA ranged from 628 to 1040 mg/L in *C. arabica* infusions. Narita and Inouye [2] reported that the highest CGA content was found in *C. arabica* from Brazil. It was found highest in green coffee beans of *C. arabica*, ranging between 3.40–14.4% *w*/*w* dry matters. The experimental previous studies of caffeine were mixed and the correlations between the levels of caffeine with the degree of roasting varied from negative to constant and positive. The majority of these studies indicated that as the degree of roasting increases, the level of caffeine increases, reaching the maximum in the light and medium roast coffee, then the caffeine levels start to decline in the dark roast coffee. It is anticipated that increasing the temperature can reduce the water content in the coffee beans and therefore can help release the volatile compounds (e.g., caffeine) from coffee; indeed, the caffeine levels were reduced significantly compared to the light and medium roast coffee after increasing the temperature to higher limits (dark roast) [13,14,15].

CGA has an important role in determining coffee beans’ quality and beverage taste and is a key contributor to the radical scavenger activity of coffee brews [16,17]. The previous studies demonstrated that there is a loss of CGA during roasting. The higher the roasting degree, the lower the content of CGA [15]. As expected, in our study, a large variability was observed in the CGA content of the coffee samples which ranged between 5.43 to 0.9% *w*/*w* (543.23 to 90.53 mg/L), in green beans throughout dark roasted beans, which represents an approximate six-fold reduction. These findings suggest that these coffee beans were roasted under high temperatures and moist conditions. Similarly, Budryn et al. [16] found that the concentration of CGA in dark roasted *C. arabica* was 5-fold lesser than in green beans. The reduced content of CGA by roasting was explained by previous studies. It was found that the increased levels of free caffeic acid were attributed to the hydrolysis of CGA during the curing of this type of specialty coffee by exposing them to moisture during the roasting process [12,18,19].

The CGA richest coffees were those that also registered the highest TEAC. The results were in good agreement with the previous reports about the antioxidants of coffee [20,21]. These findings indicate that only CGA (rather than the total phenolics) is responsible for the antioxidant activity associated with coffee consumption.

It was previously found that roasting level would contribute more to changes in CGA content than the influence of the geographical origin [22]. As expected, the subgroup with the highest levels of CGAs among all different types of roasted coffee (green, light, medium, and dark) was found in the Ethiopian coffee, at 4.08% (488 mg/L), while the lowest was found in Indian coffee, at 1.48% (148 mg/L). On the other hand, the subgroup with the highest levels of caffeine among all different types of roasted coffee (green, light, medium, and dark) was found in the Indian coffee, at 2.54% *w*/*w*, while the lowest was found in Columbian coffee, at 1.60% *w*/*w*.

Among the same samples, caffeine content ranged from 1.67 to 2.03% *w*/*w* (166.72 to 203.63 mg/L), in green coffee to medium roasted coffee beans, while a slight reduction was detected in further roasting samples. Similar results were obtained in a study by Trandafir et al. [23], which detected caffeine contents of some commercial coffees available on the Romanian market. It found that caffeine content ranged from 1.89 to 3.05% *w*/*w*. A large variability was observed in CGA content of the investigated coffee samples, which ranged between 0.6 and 2.32% *w*/*w*. Farah et al. [24], reported caffeine content in regular coffees of around 2.54–3.33% *w*/*w*, which matches the results in several studies [10,15], while some studies showed no correlation between the caffeine content and the degree of roasting [25].

## 4. Experimental Part

### 4.1. Materials and Reagents

Caffeine standard, anhydrous, extra pure, was purchased from AZ Chem. (Thunder Bay, ON, Canada); its purity was certified to be 99.8%. CGA standard, extra pure, was purchased from Tokyo Chemical Industry CO. LTD, Japan; its purity was certified to be 98.0%. Methanol, Chromosolv. for HPLC ≥99.9%, was purchased from Honeywell Research Chemicals (Muskegon, MI, USA). Water, in compliance to the specifications of USP, BP, and EP grades, was purchased from LABCHEM, (Zelienople, PA, USA). Acetonitrile (HPLC grade) was purchased from ALPHA Chemika (Mumbai, India). Formic acid (96%, ACS grade) was purchased from TEDIA (Fairfield, OH, USA).

### 4.2. Sample Preparation and Extraction

A total 52 samples of ground coffee beans (*Coffea Arabica*), which consisted of light-roasted (14 samples), medium-roasted (11 samples), dark-roasted (16 samples), and green coffee (11 samples) from various origins, were acquired from several stores in Jordan. The extraction of coffee samples was performed according to the extraction procedure that was described by Hernandez et al. [26], but with minor modifications. All coffee samples were extracted using hot water at 75–85 °C at a ratio of 1:100 (coffee-to-solvent ratio). Next, the coffee samples were ultra-sonicated for 5 min in order to homogenize the coffee solutions (ultrasonic bath, OVAN, Barcelona, Spain). Afterwards, the coffee samples were centrifuged for fifteen minutes at a speed of 7900× *g* using a laboratory centrifuge machine (MPW-260R, Warsaw, Poland). Then, the coffee extracts were filtered using Whatman No. 2 filter paper. Lastly, the coffee extracts were preserved in the freezer at a temperature of −20 °C until the analysis day.

### 4.3. Instruments and Conditions

HPLC instrumental setup comprised products of Hitachi Technologies (Tokyo, Japan). All analyses were performed in an air-conditioned laboratory (18 ± 2 °C). The measurements were performed on a VWR-Hitachi Elite LaChrom system (Pump L-2130, Autosampler L-2200, Column oven L-2300, UV Detector L-2455, VWR, (Tokyo, Japan), equipped with a (30 cm × 4 mm × 5 µm) Supelcosil LC-18-DB column (SUPELCO), flow rate of 1.0 mL/min, and UV detection was performed at 274 nm for caffeine and 330 nm for CGA. An organizer L-2000 system (Tokyo, Japan), with a diode array detector (VWR Hitachi) was controlled by EZChrom Elite (Chromatography Data System, version 3.3.2 SP1, Scientific Software Inc., Agilent, Santa Clara, CA, USA) software. All injections were performed using HPLC syringe filters (0.45 µm).

### 4.4. Preparation of Mobile Phase

For caffeine: The mobile phase was prepared by mixing water/methanol (60:40, *v*/*v*). The mobile phase was degassed by ultrasonic vibrations for 20 min prior to use.

For CGA: The mobile phase was prepared by mixing 0.1% formic acid/acetonitrile (85:15, *v*/*v*). The mobile phase was degassed by ultrasonic vibrations for 20 min prior to use.

### 4.5. Standard Solutions

The standard stock solution of caffeine was prepared by transferring 0.1002 g of the caffeine standard (equivalent to 100 mg of caffeine standard) into a 100 mL volumetric flask, and then dissolving it in the eluent to prepare a 1000 ppm concentration. The standard stock solution of CGA was prepared by transferring 0.1020 g of the CGA standard (equivalent to 100 mg of CGA standard) into a 100 mL volumetric flask, and then dissolving it in methanol to prepare a 1000 ppm concentration.

### 4.6. Calibration Curves

For HPLC, calibration standard solutions for caffeine (10, 50, 100, 200, 500, 1000 ppm) were prepared from the standard stock solution (1000 ppm) by appropriate dilution processes using the mobile phase. Calibration standard solutions for CGA (10, 50, 100, 200, 500, 1000 ppm) were prepared from the standard stock solution (1000 ppm) by appropriate dilution processes using methanol. The peak area obtained for each caffeine concentration was plotted versus concentration, and the regression equation was computed.

### 4.7. Method Development

The total run-time of the HPLC analysis for both compounds ranged from 8–9 min. The correlation coefficients (r) were calculated from external-standard calibration curves and the values were 0.9998 for caffeine and 0.9999 for CGA, respectively. The concentration was found to be proportional to the area, and the response of the detector was determined to be linear over the range of 10 to 1000 mg/L. The retention time and shape of the caffeine and CGA peaks between the standards and samples chromatograms showed a precise similarity and no interference was found. Consequently, the identification of the caffeine and CGA in the coffee samples was confirmed. The content of caffeine and CGAs in the coffee samples (mg/L) were determined using the calibration curve, and the percentage of these bioactive compounds were calculated by taking the mass of caffeine calculated from the calibration curve.

### 4.8. Statistical Analysis

The measurements of the coffee sample were performed in triplicates. The results were expressed as mean ± standard deviation for all the replicate measurements. The data obtained were statistically analyzed by using SPSS software (version 25, Chicago, IL, USA). The data were also evaluated using one-way ANOVA to test the significance differences in the mean values of caffeine and CGA levels obtained by the HPLC method.

## Figures and Tables

**Table 1:** The average concentration (mg/L) for caffeine and chlorogenic acid (CGA) content in coffee beans (*n* = 52) with different roasting degrees, regardless of geographical origin.

Coffee Type	Roasting Temperature (°C)	CGA (Average ± SD)		Caffeine (Average ± SD)	
		(mg/L)	(%)	(mg/L)	(%)
Green Coffee (*n* =11)	---	543.23 ± 8.916 ^a^	5.43 ± 0.089 ^a^	166.72 ± 5.08	1.67 ± 0.051
Light Roast (*n* = 14)	155–165	270.93 ± 10.759 ^a^	2.71 ± 0.108 ^a^	196.35 ± 6.67	1.96 ± 0.067
Medium Roast (*n*= 11)	175–185	187.45 ± 9.05 ^a^	1.87 ± 0.091 ^a^	203.63 ± 3.158	2.03 ± 0.032
Dark Roast (*n* = 16)	205–215	90.53 ± 12.97 ^a^	0.91 ± 0.130 ^a^	189.85 ± 5.81	1.90 ± 0.058

Values followed by the letter ^a^ are statistically significant (*p* < 0.05).

## Data Availability

Not applicable.

## Supplementary Materials

The following are available online. Table S1: The Average concentration (%), SD, Min, Max, Median for caffeine and chlorogenic acid content in coffee beans (*n* = 52) obtained from the Jordanian market; Figure S1: HPLC-DAD chromatogram of caffeine standard dissolved in MeOH:H_2_O [40:60] (Rt = 3.84 min, λ = 273 nm); Figure S2: HPLC-DAD chromatogram of caffeine extract sample (Rt = 3.90 min, λ = 273 nm); Figure S3: HPLC-DAD chromatogram of CGA standard dissolved in MeOH (Rt = 4.98 min, λ = 330 nm); Figure S4: HPLC-DAD chromatogram of CGA extract sample (Rt = 5.09 min, λ = 330 nm).

## Sample Availability

Samples of the standard compounds are available from the authors.
