# Supplementary material for: Quantification of Caffeine and Chlorogenic Acid in Green and Roasted Coffee Samples Using HPLC-DAD and Evaluation of the Effect of Degree of Roasting on Their Levels
Source: Molecules. 2021 Dec 11;26(24):7502. doi: 10.3390/molecules26247502 (PMC8705492; doi:10.3390/molecules26247502)
Supplement: Supplementary file 1 [file molecules-26-07502-s001.zip › molecules-1463274-SM update.pdf]

molecules

Article

Quantification of Caffeine and Chlorogenic Acid in Green and Roasted Coffee Samples Using HPLC-DAD and Evaluate the Effect of Degree of Roasting on Their Levels

Shady Awwad [1,*], Reem Issa [2,*], Lilian Alnsour [2], Dima Albals [3] and Idrees Al-Momani [4]

[1] Department of Pharmaceutical Chemistry and Pharmacognosy, Applied Science Private University, Amman 11931, Jordan
[2] Department of Pharmaceutical Sciences, Pharmacological and Diagnostic Research Center (PDRC), Faculty of Pharmacy, Al-Ahliyya Amman University, Amman 19328, Jordan; l.alnsour@ammanu.edu.jo
[3] Department of Medicinal Chemistry and Pharmacognosy, Faculty of Pharmacy, Yarmouk University, Irbid 21163, Jordan; dimabals@yu.edu.jo
[4] Department of Chemistry, Faculty of Science, Yarmouk University, Irbid 21163, Jordan; imomani@yu.edu.jo
* Corresponding: sh_awwad@asu.edu.jo (S.A.); r.issa@ammanu.edu.jo (R.I.)

Table S1: The Average concentration (%), SD, Min, Max, Median for caffeine and chlorogenic acid content in coffee beans (n=52) obtained from the Jordanian market.

**Caffeine**						
**Data**	**Avg. Conc. (%)**	**SD**	**Min**	**Max**	**Median**	**N**
All	1.90	0.55	0.99	3.29	1.85	52
Brazil	2.03	0.48	0.99	2.71	2.01	15
Colombia	1.60	0.36	1.13	2.83	1.55	17
Ethiopia	2.14	0.51	1.43	2.83	2.03	6
India	2.54	0.92	1.35	3.29	2.76	4
Kenya	1.72	0.46	1.21	2.33	1.49	9
Saudi Arabia	2.47	---	2.47	2.47	2.47	1
**Chlorogenic Acid**						
All	2.54	1.95	0.15	7.45	2.34	52
Brazil	1.91	1.64	0.18	5.46	1.51	15
Colombia	2.80	2.15	0.35	7.45	2.52	17
Ethiopia	4.08	2.06	0.62	5.84	5.06	6
India	1.48	1.13	0.15	2.91	1.43	4
Kenya	2.36	1.95	0.50	6.09	1.71	9
Saudi Arabia	3.91	---	3.91	3.91	3.91	1

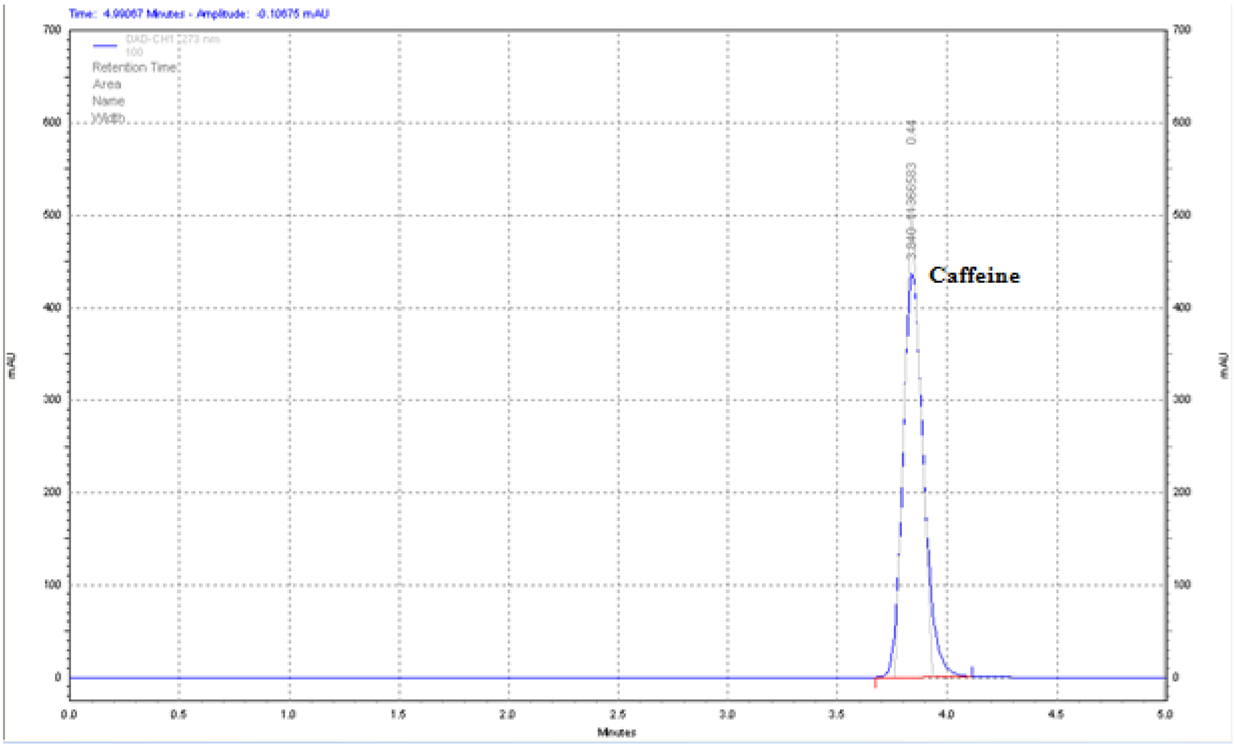

Figure S1: HPLC-DAD chromatogram of caffeine standard dissolved in MeOH:H_2O [40:60] (Rt = 3.84 min, λ = 273 nm)

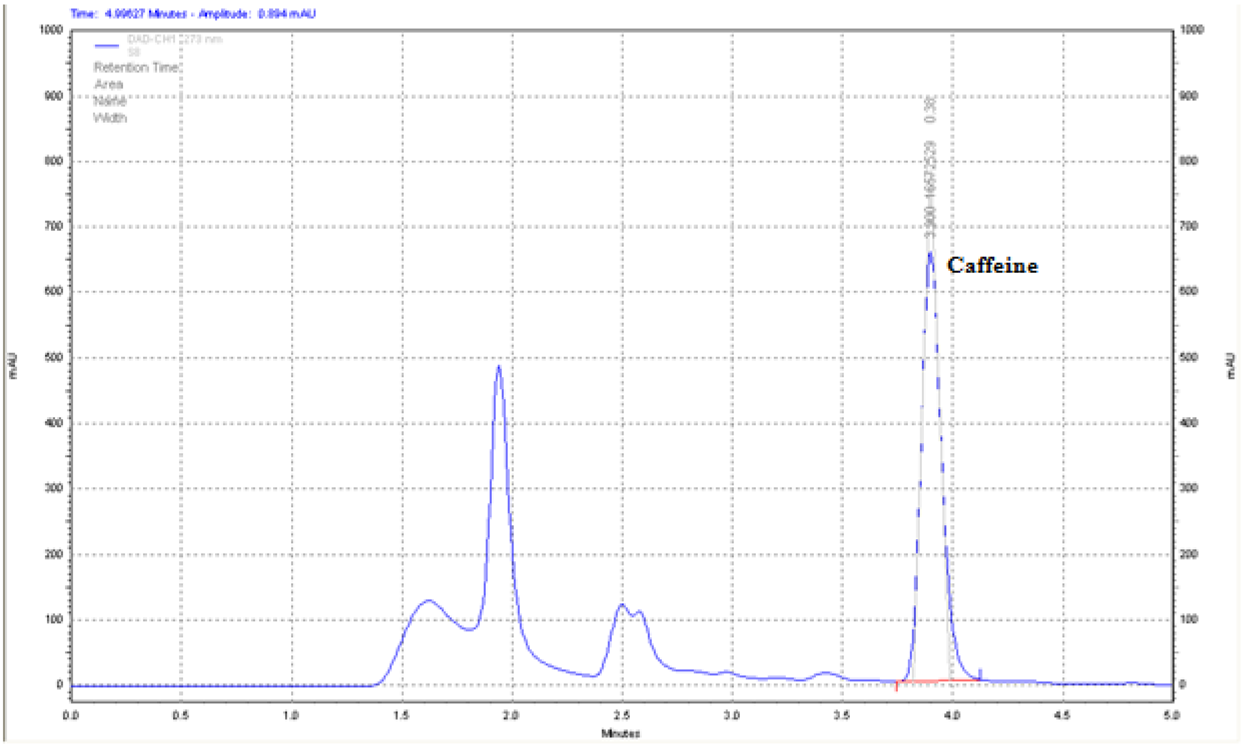

Figure S2: HPLC-DAD chromatogram of caffeine extract sample (Rt = 3.90 min, λ = 273 nm)

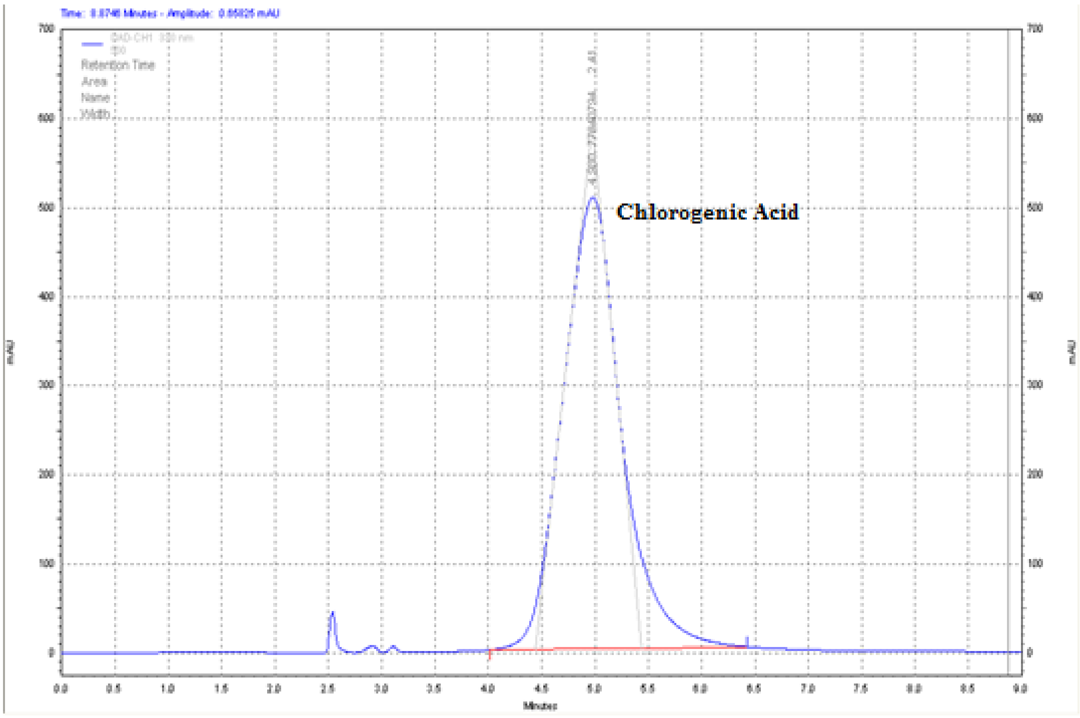

Figure S3: **HPLC-DAD chromatogram of CGA standard dissolved in MeOH (Rt = 4.98 min, λ = 330 nm)**

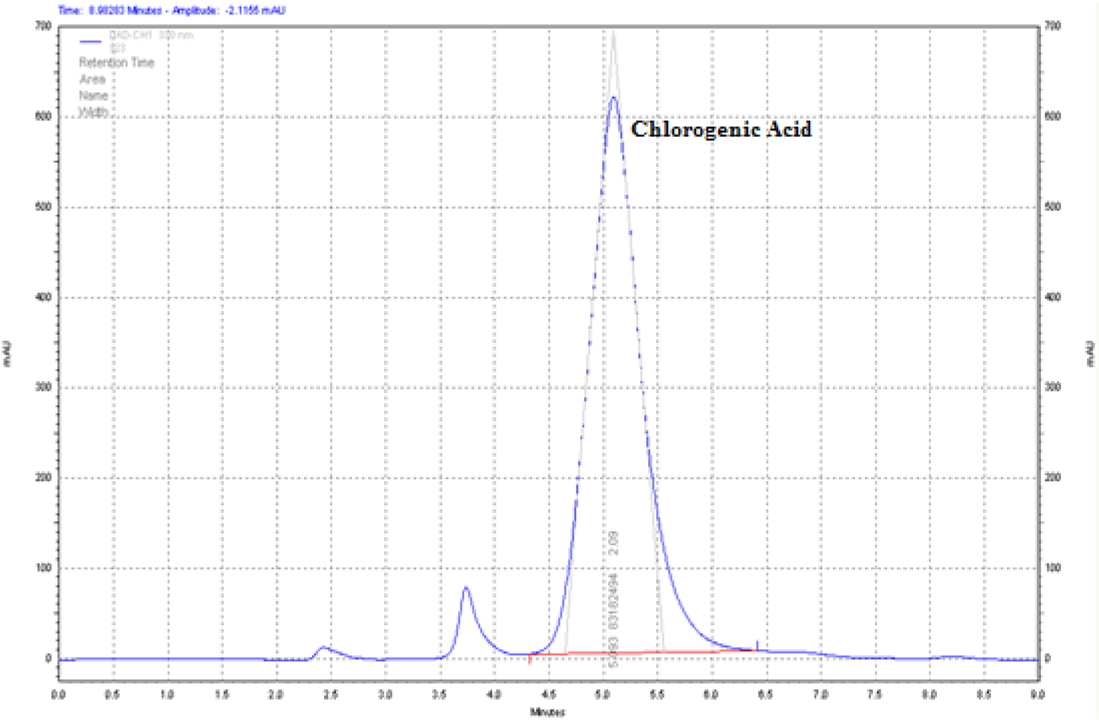

Figure S4: **HPLC-DAD chromatogram of CGA extract sample (Rt = 5.09 min, λ = 330 nm)**
